# Diclofenac Loaded Biodegradable Nanoparticles as Antitumoral and Antiangiogenic Therapy

**DOI:** 10.3390/pharmaceutics15010102

**Published:** 2022-12-28

**Authors:** Gerard Esteruelas, Eliana B. Souto, Marta Espina, María Luisa García, Marta Świtalska, Joanna Wietrzyk, Anna Gliszczyńska, Elena Sánchez-López

**Affiliations:** 1 Department of Pharmacy, Pharmaceutical Technology and Physical Chemistry, Faculty of Pharmacy and Food Sciences, University of Barcelona, 08007 Barcelona, Spain; 2 Institute of Nanoscience and Nanotechnology (IN2UB), University of Barcelona, 08007 Barcelona, Spain; 3 Department of Pharmaceutical Technology, Faculty of Pharmacy, University of Porto, 4050-313 Porto, Portugal; 4 REQUIMTE/UCIBIO, Faculty of Pharmacy, University of Porto, 4050-313 Porto, Portugal; 5 Department of Experimental Oncology, Ludwik Hirszfeld Institute of Immunology and Experimental Therapy, Polish Academy of Sciences, Weigla 12, 53-114 Wrocław, Poland; 6 Department of Food Chemistry and Biocatalysis, Faculty of Biotechnology and Food Science, Wrocław University of Environmental and Life Sciences, Norwida 25, 50-375 Wrocław, Poland

**Keywords:** diclofenac, nanoparticles, PLGA, anti-inflammatory, drug delivery, anti-angiogenesis, antitumoral

## Abstract

Cancer is identified as one of the main causes of death worldwide, and an effective treatment that can reduce/eliminate serious adverse effects is still an unmet medical need. Diclofenac, a non-steroidal anti-inflammatory drug (NSAID), has demonstrated promising antitumoral properties. However, the prolonged use of this NSAID poses several adverse effects. These can be overcome by the use of suitable delivery systems that are able to provide a controlled delivery of the payload. In this study, Diclofenac was incorporated into biodegradable polymeric nanoparticles based on PLGA and the formulation was optimized using a factorial design approach. A monodisperse nanoparticle population was obtained with a mean size of ca. 150 nm and negative surface charge. The release profile of diclofenac from the optimal formulation followed a prolonged release kinetics. Diclofenac nanoparticles demonstrated antitumoral and antiangiogenic properties without causing cytotoxicity to non-tumoral cells, and can be pointed out as a safe, promising and innovative nanoparticle-based formulation with potential antitumoral effects.

## 1. Introduction

Regardless of type, cancer is still considered as the second leading cause of death worldwide, representing a serious public health problem [1]. The changes of a healthy cell into a tumoral one follow a slow pathway that requires specific abilities (e.g., resistance against cell death, evasion of the immune system, induction of angiogenesis and/or activation of metastasis), all known as hallmarks of cancer [2,3,4]. According to the latest World Health Organization (WHO) report, it is estimated that by 2040 the number of cancer patients will increase 47% in comparison to 2020, which will result in 16 million deaths [5]. Therefore, there is an urgent need to obtain antitumoral therapies able to treat cancerous cells without affecting healthy tissues.

Diclofenac (DCF) is a drug from the non-steroidal anti-inflammatory (NSAID) family. In normal clinical practice it is used to relieve pain or as antipyretic, but it has its own characteristics that make it of special interest for antitumoral therapy, such as the ability to inhibit COX-2, or some prostaglandins, or the ability to act on the immune system in the angiogenesis process [6,7,8]. However, DCF also possesses some adverse effects, such as gastric toxicity. In recent years, modified release systems with site-specific targeting features have been exploited for drug delivery, in particular for cancer treatment, with the purpose of reducing the amount of drug required for the treatment attributed to the specific delivery of the drug in the site of action for a longer time, reducing the side effects. In addition, delivery systems may also improve drug stability and increase permeability and bioavailability [9,10,11]. Therefore, encapsulation of drugs such as DCF into biodegradable polymeric nanoparticles (NPs) is proposed, to enhance the drug’s therapeutic activity and half-life in the site of action, reducing the systemic drug exposure and thus decreasing adverse effects [12,13,14,15].

Poly (lactic-co-glycolic acid) (PLGA) is amongst the most commonly recommended polymers for the development of drug delivery systems, and for NPs in particular, due to its biocompatibility and biodegradability. PLGA is categorised as a generally regarded as safe (GRAS) substance by the European Medicines Agency (EMA) and Food and Drug Administration (FDA), besides showing the ability to act as a carrier targeting the drug to the site of action [16,17].

In the present study, we describe the pharmaceutical development of PLGA NPs for the loading of DCF, and their physicochemical characterization with the purpose of being used for antitumoral therapy. Formulations were optimized by factorial design, and the optimal DCF-loaded NPs were characterized with respect to drug-polymer interaction, release profile and safety, as well as antitumoral and antiangiogenic properties.

## 2. Materials and Methods

### 2.1. Materials

Diclofenac sodium and ethyl acetate were purchased from Sigma-Aldrich (St. Louis, MO, USA); PLGA Resomer^®^ 50:50 503H was acquired from Boehringer Ingelheim (Ingelheim am Rhein, Germany). Water filtered through a Millipore^®^ MilliQ system was used for all the experiments. All other reagents used were of analytical grade.

### 2.2. Nanoparticles Preparation

A modified double emulsion procedure described elsewhere [18,19,20] was used for the production of DCF NPs. Briefly, a predetermined amount of PLGA (defined in the DoE) was dissolved in 2 mL of ethyl acetate to form the organic phase (o). The inner aqueous phase (w_1_) was prepared by dissolving DCF in 2.5 mL of MilliQ water. This inner water phase was dispersed in the organic phase to obtain the primary emulsion (w_1_/o) by applying ultrasonic energy over 30 s. The secondary emulsion (w_1_/o/w_2_) was then obtained by mixing the primary w_1_/o emulsion into the external aqueous phase based on 1.5 mL of MilliQ water containing Lutrol F68 (2.0 mg·mL^−1^) by ultrasonic energy. Finally, 1 mL of Lutrol (0.02 mg·mL^−1^) was added dropwise under magnetic stirring, and the organic solvent was evaporated overnight [20].

### 2.3. Design of Experiments

A factorial design was implemented to achieve the optimal formulation of DCF NPs, using a matrix generated by StatGraphics Centurion XVI.I. The design of experiments (DoE) was developed to analyze the effects of the independent variables (concentration of DCF, PLGA and P188) on the dependent variables (mean particle size (Z_av_), polydispersity index (PI), zeta potential (ZP) and encapsulation efficiency (EE)) [21,22]. Each factor was studied at five different levels (Table 1) and the responses were modelled by means of the full second-order polynomial Equation (1):

(1) $$Y=\beta_{0}+\beta_{1}X_{1}+\beta_{2}X_{2}+\beta_{3}X_{3}+\beta_{11}X_{1}^{2}+\beta_{22}X_{2}^{2}+\beta_{33}X_{3}^{2}+\beta_{12}X_{1}X_{2}+\beta_{13}X_{1}X_{3}+\beta_{23}X_{2}X_{3}$$

where *Y* stands for the measured response, *β*_0_ to *β*_23_ stand for the regression coefficients and *X*_1_, *X*_2_ and *X*_3_ stand for the studied factors [23].

### 2.4. Physicochemical Characterization

The Z_av_ and PI of DCF NPs were determined by dynamic light scattering after 1:10 dilution using a ZetaSizer NanoZS (Malvern Instruments, Worcestershire, United Kingdom). The particle electrophoretic mobility was used to determine ZP, applying a combination of Laser doppler velocimetry and phase analysis light scattering (PALS). Prior to measurements, each sample was diluted followed by the triplicate analysis in 10 mm diameter cells and disposable capillary cells DTS1070 (Malvern Instruments, Worcestershire, United Kingdom), for Z_av_ and ZP respectively, at 25 °C [18,24,25].

In order to quantify the amount of DCF encapsulated into the NPs, the EE was determined indirectly by measuring the non-loaded DCF in the dispersion medium as performed elsewhere [26,27]. Filtration-centrifugation was applied at 15,000 rpm for 15 min (Beckman Optima^®^ Ultracentrifuge, Brea, CA, USA) to separate non-loaded DCF from the NPs. Then, supernatant was used to calculate the EE according to Equation (2):

(2) $$\mathrm{EE}\left(\%\right)=\frac{\mathrm{Total}\;\mathrm{amount}\;\mathrm{of}\;\mathrm{DCF}-\mathrm{Free}\;\mathrm{amount}\;\mathrm{of}\;\mathrm{DCF}}{\mathrm{Total}\;\mathrm{amount}\;\mathrm{of}\;\mathrm{DCF}}\cdot 100$$

The amount of DCF in the aqueous phase was quantified by a reverse-phase high-performance liquid chromatography (RP-HPLC) method [28]. Briefly, samples were injected in a HPLC Waters 2695 (Waters, Milford, MA, USA) using an ODS Hypersil C_18_ column (5 µm, 10 × 4.6 cm, analytical Tracer). A diode array detector Waters^®^ 2996 at a wavelength of 254 nm was used to quantify the DCF and data were processed using Empower 3^®^ Software (Waters, Milford, MA, USA). Isocratic conditions were applied consisting on two mobile phases (50:50): an organic phase (made of methanol and acetonitrile 50:50) and an aqueous phase (0.02 M sodium acetate at pH = 7.0), at a flow rate of 0.5 mL/min. DCF standards (0.5–500 µg/mL) were prepared and dissolved in MilliQ water.

### 2.5. Morphological Characterization

Morphology of DCF NPs was performed by Transmission Electron Microscopy (TEM), performed on a JEM 1010 microscope (JEOL, Akishima, Japan). DCF NPs were placed on copper grids previously activated with UV light and subjected to negative staining with 2% uranyl acetate [21,29].

### 2.6. Interaction Studies

For the interaction studies, DCF NPs were firstly ultracentrifuged (Beckman Optima^®^ Ultracentrifuge, CA, USA) at 45,000 rpm and 4 °C for 60 min. The obtained pellet was dried using N_2_ and pulverized to obtain a powder.

X-ray diffraction (XRD) was carried out to study the amorphous-crystalline state of the optimized formulation. The sample was placed between two polyester films and then subject to CuK radiation (45 kV, 40 mA, = 1.5418 Å), range of 2θ, from 2° to 60°step size of 0.026 and a time of 200 s for step measurement [21,30].

In order to analyze the covalent bonds, Fourier Transform Infrared (FTIR) analysis was performed using a Thermo Scientific Nicolet iZ10 (Madison, WI, USA) with an ATR diamond and DGTS detector (scanned range from 525 to 4000 cm^−1^ and spectral of 4 cm^−1^ and 32 scans) [23].

### 2.7. γ-Irradiation Sterilization

In order to remove microbial contamination, DCF NPs were sterilized using 25 kGy of ^60^Co as γ-irradiation source (Aragogamma, Barcelona, Spain) [1]. This corresponds to a standard dose to sterilize pharmaceutical products when bioburden is not known. Physicochemical properties of DCF NPs were evaluated before and after irradiation [31].

### 2.8. In Vitro Drug Release

An in vitro drug release was performed by direct dialysis using dialysis bags to evaluate the kinetic profile of DCF from the polymeric matrix. For this purpose, phosphate saline buffer (PBS) at 37 °C was used as dialysis medium. At predetermined time intervals for 24 h, 0.3 mL were removed and the volume was replaced with 0.3 mL of PBS, this experiment was carried out by triplicate and complying with the DCF solubility sink conditions in the Dialysis medium [7,31]. After the analysis, the amount of DCF released was determined by HPLC and the data was analyzed with the GraphPad Prism 7 software.

In addition, physicochemical parameters (Z_av_, PI, ZP and EE) were also measured every 24 h after the preparation of NPs at 37 °C in order to confirm their stability at physiological temperature.

### 2.9. Short-Term Stability

The stability of DCF-NPs stored at different temperatures (4, 25 and 38 °C) was studied by measuring the backscattering and transmission profiles using Turbiscan^®^ Lab (Iesmat, Madrid, Spain) [32]. Therefore, a glass measurement cell was filled with 20 mL of sample and a light source corresponding to a pulsed near infrared (λ = 880 nm) was applied. It was received by a backscattering detector (at 45° from the incident beam) as well as a transmission detector. Backscattering and transmission data were acquired for two months, carrying out readings during 24 h at intervals of 1 h. Moreover, physicochemical parameters (Z_av_, PI, ZP and EE) were also measured monthly.

### 2.10. Antiangiogenic Capacity

In order to evaluate the antiangiogenic effects of DCF NPs, the modified Choriolantoic Membrane (CAM) test was used. For this purpose, fertilized chicken eggs were incubated at 37 °C and 85% humidity. After 3 days under incubation, a window was opened on the side of the egg in the shell, and after 24 h, 40 µL of the formulation were inoculated to the exposed CAM. Afterwards the CAM was subsequently hermetically sealed, incubated and evaluated at 48 h after administration, always maintaining aseptic conditions [33,34].

After 48 h, the CAMs were fixed by adding 4% paraformaldehyde for 24 h at 4 °C. Following this procedure, membranes were removed and observed with a binocular loupe [33]. Subsequently, images were process and the density of vessels in the CAM was measured automatically using ImageJ vessel analysis plugin [35].

### 2.11. In Vitro Cytotoxicity Assay

#### 2.11.1. Cell Lines

Human biphenotypic B myelomonocytic leukemia (MV4-11) and human normal breast epithelial cells (MCF-10A) were obtained from American Type Culture Collection (Manassas, VA, USA) whereas lines A549 (lung cancer) and MCF-7 (breast cancer) from European Collection of Authenticated Cell Cultures (Salisbury, UK). The fifth studied cell line MDA-MB-468 (breast cancer) was obtained from Leibniz Institute DSMZ-German Collection of Microorganisms and Cell Cultures (Braunschweig, Germany) [36,37].

Established in vitro cell lines were maintained in the Institute of Immunology and Experimental Therapy, Wroclaw, Poland. RPMI 1640 medium (IIET PAS, Poland) with 1.0 mM sodium pyruvate (only MV4-11) and 10% (MV4-11) or 20% (MDA-MB-468) fetal bovine serum (FBS) (all from Merck, Darmstadt Germany) was used for MV4-11 and MDA-MB-468 cells. In case of cells of line A549 RPMI 1640+Opti-MEM (1:1) (IIET PAS, Poland and Gibco, UK) supplemented with 5% fetal bovine serum was used as the medium (Merck, Germany) whereas breast cancer cells line MCF-7 were cultured in Eagle medium (IIET PAS, Poland) containing 8 µg/mL of insulin and 1% of MEM non-essential amino acid (all Merck, Germany). Medium HAM’S F-12 medium (Corning) supplemented with 10% horse serum (Gibco), 20 ng/mL EGFh, 10 µg/mL insulin, 0.5 µg/mL hydrocortisone and 0.05 mg/mL cholera toxin from Vibrio cholerae (all from Merck, Germany) was used for MCF-10A cells [1]. Moreover, all used for cells growth media were supplemented with 2 mM L-glutamine (Merck, Germany), 100 units/mL penicillin, (Polfa Tarchomin S.A., Warszawa, Poland) and 100 µg/mL streptomycin (Merck, Germany). Growth of cells was carried out at 37 °C in the 5% CO_2_ humidified atmosphere [38].

#### 2.11.2. Determination of Antiproliferative Activity

Solutions of DCF in water, empty NPs and DCF NPs (1.2 mg/mL) were prepared and then diluted with culture medium to reach the final concentrations. Twenty-four hours prior to the addition of DCF and fabricated nanoformulation, selected cell lines were plated in 96-well plates (Sarstedt, Germany) at a density of 1 × 10^4^ or 0.5 × 10^4^ (A549) cells per well. Tested solutions at the final concentrations (100, 10, 1 and 0.1 μg/mL concentration) were added to the culture medium and the cells were continued for 72 h.

For evaluation of the cytostatic effect of DCF, fabricated DCF NPs and empty NPs the MTT assay for leukemia cells (MV4-11) or SRB assay for adherent cells were performed according to the previously described procedure [39]. The results of antiproliferative activity in vitro were expressed as IC_50_ (concentration of studied compounds in μg/mL that inhibits the proliferation of 50% of cells in comparison to control untreated cells). IC_50_ values were calculated for each experiment separately using Prolab-3 system based on Cheburator 0.4 software [40]. Each concentration of each compound was tested in triplicate in a single experiment, which was then repeated 3–5 times.

### 2.12. Statistical Analysis

In order to evaluate the statistical significance of the results, to compare two groups Students *t*-test was performed, while for multi-group comparison a two-way ANOVA followed by Tukey post hoc test was performed using GraphPad Prism 7. All the data are presented as the mean ± S.D.

## 3. Results

### 3.1. Design of Experiments

A composite central factorial design was implemented to evaluate the effect of DCF, PLGA and P188 concentrations on the physicochemical properties of DCF NPs. The response parameters and their magnitudes for each of the 16 experiments are given in Table 1. Due to DCF high water solubility, the double emulsion method was used to prepare DCF NPs [20].

Considering that the Z_av_ of DCF NPs constitutes a critical parameter for crossing biological barriers and going unnoticed by the immune system, it is necessary to obtain NPs below 200 nm.

**Figure 1:**
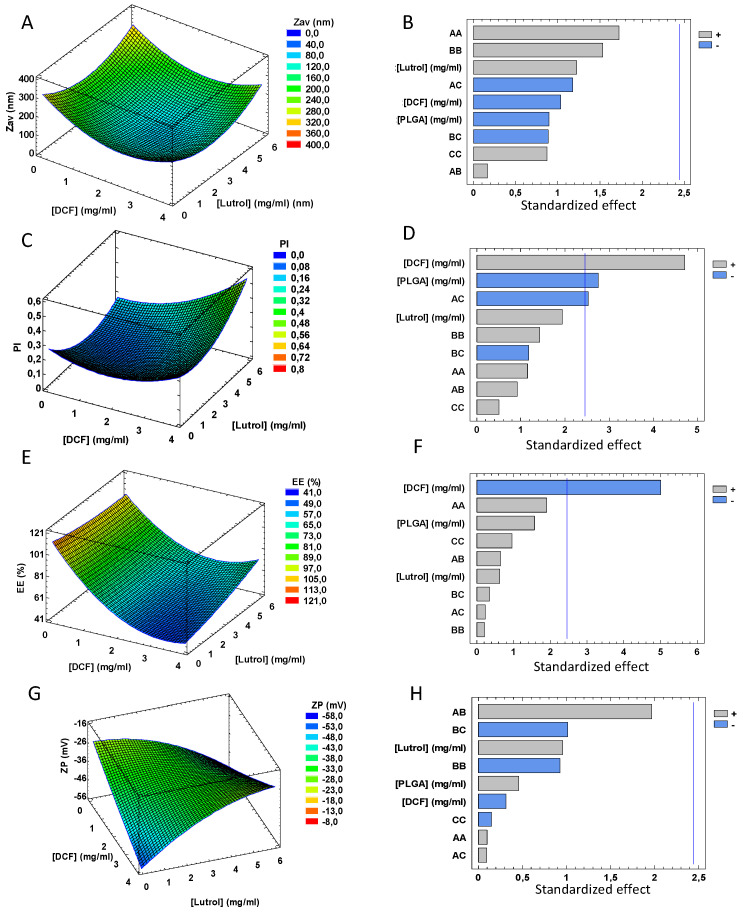
Results of the DoE of DCF NPs. (**A**,**C**,**E**,**G** correspond to surface responses and **B**,**D**,**F**,**H** correspond to Pareto diagram). (**A**) Surface response of Z_av_ at a fixed concentration of PLGA (16 mg/mL), (**B**) Standardized Pareto diagram of Z_av_, (**C**) Surface response of PI at a fixed concentration of PLGA (16 mg/mL), (**D**) Standardized Pareto diagram of PI, (**E**) Surface response of EE at a fixed concentration of PLGA (16 mg/mL), (**F**) Standardized Pareto diagram of EE, (**G**) Surface response of ZP at a fixed concentration of PLGA (16 mg/mL) (**H**) Standardized Pareto diagram of ZP.

According to the results obtained, regarding Z_av_ of DCF NPs, none of the variables studied was statistically significant. However, it was shown that at higher concentrations of PLGA and DCF, DCF NPs follow a trend towards smaller sizes, probably due to the fact that in these conditions the NPs are more compact [41]. Moreover, since Lutrol is a non-ionic surfactant used as an emulsifier, it is also important to determine the optimal concentration [21,42,43]. Through the design, it is clear that a concentration of 3 mg/mL decreases the coalescence, stabilizing the system as well as avoiding aggregation obtaining monodisperse systems (Figure 1a,b).

Moreover, PI is significantly influenced by the concentration of PLGA and DCF. As can be observed in Figure 1, to obtain PI lower than 0.1, high PLGA concentrations and low DCF amounts are necessary in an inversely proportional manner (Figure 1c,d). This may be due to the fact that lower DCF concentrations favor its encapsulation, as can be seen in the Pareto’s diagram of EE where the lower the concentration, the higher the EE. This may be due to a saturation of the polymeric matrix since the lower the concentration of DCF the greater EE (Figure 1e,f) [44].

For the ZP, a tendency towards obtaining higher ZP with increased DCF amount is observed (Figure 1g,h). This is relevant due to the ZP influence on the short-term stability of the NPs.

According to the data obtained from the factorial design, the optimal formulation parameters obtained are shown in Table 2. Using this formulation, Z_av_ of DCF NPs was 147.9 ± 1.1 nm, PI was below 0.1 (0.095 ± 0.015), ZP was highly negative (38.2 ± 4.4 mV) and EE was of 83.50%.

### 3.2. Morphological Characterization

**Figure 2:**
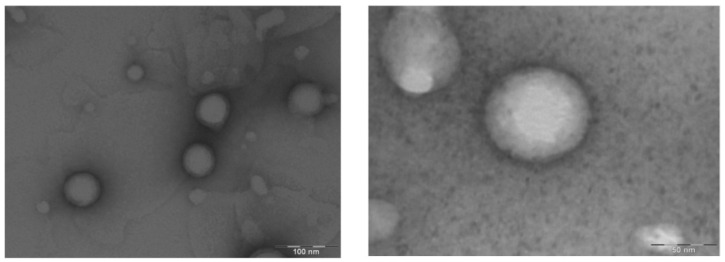
TEM images of DCF NPs observed at different magnifications.

Characterization of size and shape is of great importance to ensure that the particles are homogeneous and to determine if aggregates are present [25]. In this sense, DCF NPs observed by TEM show good size distribution that correlates with DLS measurements. It is confirmed that the sample is homogeneous, the NPs are spherical and the presence of aggregates was not observed (Figure 2).

### 3.3. Interaction Studies

**Figure 3:**
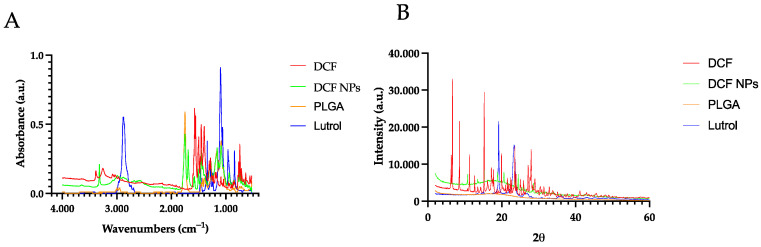
Interaction studies (**A**) FTIR spectra (**B**) XRD spectra.

Interaction studies were carried out by using FTIR and XRD. Regarding FTIR analysis, DCF showed well-defined peaks in the band of 746.63 cm^−1^ corresponding to the stretching of the C-CL bond, and in the band of 3372.06 cm^−1^ corresponding to the flexion and tension of the N-H bond of the secondary amine and 1574.64 cm^−1^ band corresponding to the bonds of the carboxylic acid [45]. Regarding the PLGA spectrum, it presented the typical bands of this polymer corresponding to the vibration of the C=O bond, the absorption of the methyl groups of lactic acid, the CH_2_ groups of glycolic acid, and the absorption of the hydroxyl group at about 3036.37 cm^−1^ and the vibrations of the C- O, = CO, C-O-C groups (Figure 3b). Moreover, in the case of DCF NPs, the spectrum bands are correlated to those obtained by DCF NP components, which may indicate that no covalent bonds were obtained [18,21,46].

XRD was carried out showing DCF crystalline structure where various characteristic peaks can be observed (15.24°, 17.84°, 19.97°, 23.56° and 27.97°) [45]. On the other hand, Lutrol, presents two peaks, mainly at 19.18° and 23.54° [47]. Moreover, DCF NPs present a very similar profile to the PLGA, but with discrete peaks that are attributed to the DCF found on the surface of the NPs (Figure 3a) [48,49].

### 3.4. In Vitro Drug Release

Previous to the in vitro drug release, stability at body temperature was carried out with the NPs. In this area, DCF NPs stored at 37 °C were stable for 72 h whereas at 96 h they already presented significant changes in both ZP and the EE.

After this examination, in vitro release profile was carried out during a period of 24 h. The release profile results show that the free DCF exhibits faster release kinetics compared to DCF NPs. After 7 h, DCF was fully released. However, DCF released from the NPs at 24 h presents a release percentage of 40% thus confirming the prolonged release caused by DCF NPs. During the first timepoints, a burst release of DCF NPs can be observed, thus corresponding to the DCF attached on the surface that was observed by XRD. Later, a prolonged release of DCF is produced, probably corresponding to the encapsulated drug. This is in accordance with other drugs encapsulated into biodegradable nanoparticles [31,50].

**Figure 4:**
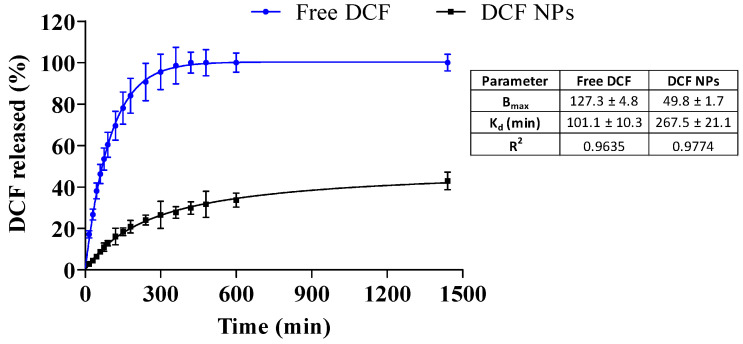
In vitro release profile of DCF NPs vs. free DCF carried out for 24 h and adjustment to a hyperbola equation.

Moreover, data was adjusted to the most common kinetic models, showing that the release profile corresponds to a hyperbola equation [1,25,51]. Both DCF NPs and free DCF data were adjusted, and the results can be observed in Figure 4. Regarding this fact, the dissociation constant (K_d_) of DCF NPs was more than double the free DCF (267.5 vs. 101.1 min), thus indicating a prolonged release [31]. In addition, after 24 h, B_max_ of DCF NPs was 49.83%, thus meaning that increased amounts of DCF will be released during longer timepoints.

### 3.5. Short-Term Stability

**Figure 5:**
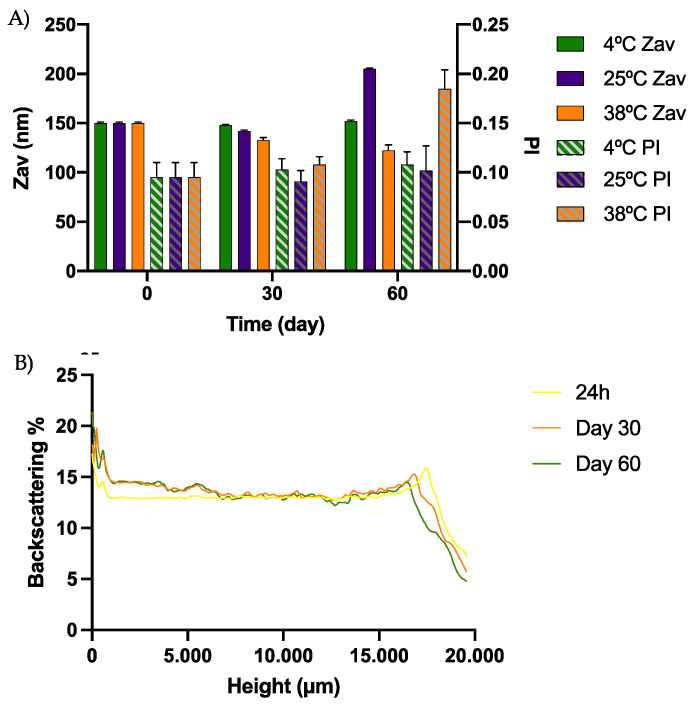

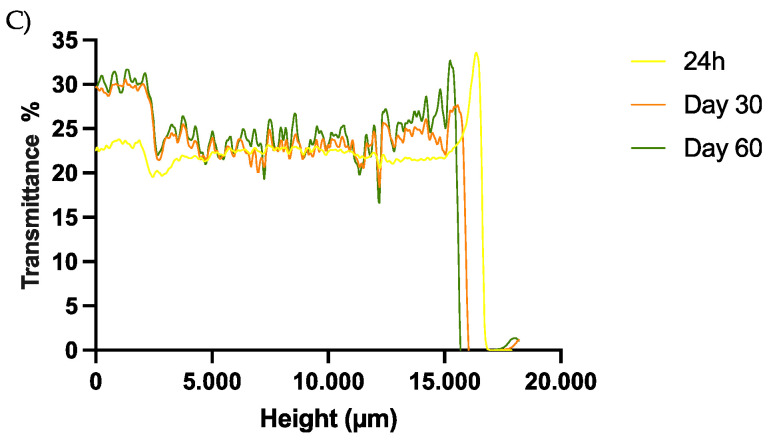
(**A**) Graphic representation of the physicochemical characterization at different storage temperatures (Z_av_ and PI at 4, 25 and 38 °C), (**B**) Turbiscan backscattering profile at 4 °C of DCF NPs, (**C**) Turbiscan Transmittance profile at 4 °C of DCF NPs.

The short-term stability studies of the optimized formulation were carried out at 4, 25 and 38 °C. As can be observed in Figure 5, these studies revealed that the suitable method of DCF NP conservation was at 4 °C, where the formulation was stable up to 60 days. No differences of more than 5% were observed in the backscattering and transmittance profile. In addition, physicochemical parameters at this temperature were also maintained (Figure 5).

In Figure S1 of the Supplementary Material, both the backscattering and transmittance profile of the DCF NPs stored at 25 °C can be observed. Modifications greater than 5% were observed at this temperature after one month, which correlates with the parameters evaluated in the physicochemical characterization, indicating an instability process at this temperature.

This is in accordance with the results obtained by other authors encapsulating hydrophilic drugs into PLGA nanoparticles, where NPs were more stable at 4 °C than at 25 °C [20]. In addition, during this period the appearance of the DCF NPs was preserved with an opaque white color and the physicochemical parameters did not present significant modifications.

As expected, at 38 °C the NPs were not stable, showing alterations in backscattering, appearance and the physicochemical parameters, probably due to the degradation of PLGA and release of the encapsulated DCF.

### 3.6. Sterilization Using γ-Irradiation

The application of γ rays is a sterilization method suitable for thermolabile compounds such as DCF NPs [27,31]. However, preservation of the physicochemical properties of the formulations after sterilization should be ensured. Therefore, Z_av_, PI, EE and ZP were measured before and after irradiation. Results are shown in Table 3 and, as can be observed, physicochemical parameters of DCF NPs were not modified after the sterilization and no significant differences were observed for any of the parameters. This results are in accordance with those obtained by other authors also using this sterilization method for polymeric nanoparticles [31].

### 3.7. Antiangiogenic Properties

**Figure 6:**
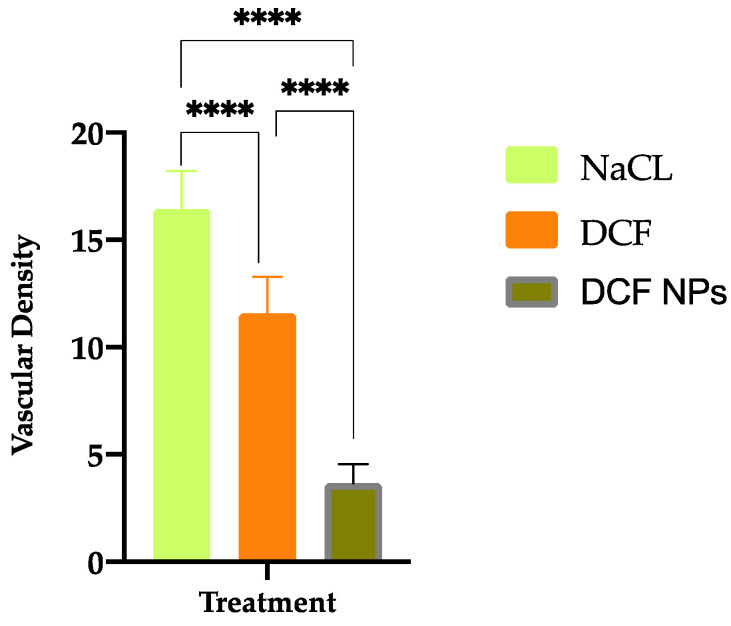
Vascular density results of the membranes after 48 h of product application (**** *p* < 0.0001).

In order to assess the anti-angiogenic capacity of DCF NPs, an in vitro test using embryonated eggs was carried out. In the extracted membranes, it was observed that NaCl control presents a greater number of ramifications of new blood vessels with respect to DCF and DCF NPs (Figure 6). Moreover, quantification of the density is shown in Figure 5. Vascular density results indicate that both DCF NPs and DCF were able to decrease the density of the blood vessels compared to the NaCl control. This correlates with studies carried out by other authors that indicate that DCF poses anti-angiogenic properties [52,53].

Both DCF NPs and DCF presented a greater capacity to inhibit the formation of new blood vessels compared to NaCl (*p* < 0.0001). Furthermore, significant differences in vascular density were also observed by comparing DCF NPs and free DCF obtaining increased anti-angiogenic effect with DCF NPs (*p* < 0.0001). This may be due to the greater penetration capacity of DCF NPs against free DCF, as well as the fact that, as demonstrated in the dialysis experiment, NPs are capable of releasing DCF in a prolonged manner.

In Figure S2 of the Supplementary Material, the images of the CAM at 0, 24 h and 48 h before extracting the membranes for their fixation can be found. It can be observed that at the initial timepoint, all have a similar vessel density, whereas afterwards different amount of blood vessels was formed depending on the treatment applied.

### 3.8. Cytotoxicity of Fabricated Formulation towards Selected Cancer Cell Lines

The cytotoxic activities of DCF and DCF NPs against leukemia (MV4-11), lung (A549) and two breast MCF-7 (ER+) and MDA-MB-468 (TNBC, triple-negative) cancer cell lines were evaluated using cellular viability assessment by MTT (MV4-11) or SRB colorimetric assays. As only 40% of DCF was released after 24 h, as in the in vitro release assay, we decided to evaluate the antiproliferative activity after 72 h, and the results are shown in Table 4.

In our study, we also examined the toxicity of DCF and DCF NPs towards the human normal breast epithelial MCF-10A cells, as well as the toxicity of empty NPs.

According to the data shown in Table 4, DCF inhibited growth of all tested cell lines (with IC_50_ in the range 23.95–37.26 μg/mL) but DCF NPs was active only towards leukemia MV4-11, lung cancer A549 and breast cancer MCF-7 cells. Determined IC_50_ values were two times higher for DCF NPs in comparison to those determined for DCF at lung (A-549) and breast (MCF-7) cancer cell lines. Moreover, empty NPs did not exhibit cytotoxic activity towards studied cell lines. DCF NPs was not active towards triple-negative breast cancer cell line MDA-MB-468 and, importantly, was not active towards non-tumoral cell lines. The latter indicates the increased safety of DCF NPs against free DCF, which correlates with fewer adverse effects provided by DCF NPs against DCF.

## 4. Conclusions

In the present study, a new formulation based on PLGA NPs was prepared, optimized, and characterized, with the ability to encapsulate DCF and able to release it in a prolonged way over time and thus be able to overcome the overcome the needs and challenges of antitumoral and anti-cancer therapies. A DoE method was used to optimize the DCF-NPs. Interaction, morphological studies and the physicochemical properties as nanoparticle size, distribution size, surface charge and entrapment efficacy were obtained and analysed, demonstrating suitable characteristics to be able to interact with the immune system and be able to fulfil the proposed objective. Finally, in vitro therapeutic efficacy studies demonstrated that DCF NPs are able to enhance the anti-angiogenic capacity of DCF, providing increased anti-angiogenic activity. Moreover, DCF NPs show anti-tumoral effects against tumoral cell lines without exerting cytotoxic effects in non-tumoral cells. Therefore, DCF NPs could constitute a potentially suitable approach for cancer therapy.

## Figures and Tables

**Table 1:** DoE matrix, levels and physicochemical characterization of the formulations.

	C_DCF_		C_Lutrol_		C_PLGA_		Z_AV_ ± SD (nm)	PI ± SD	ZP ± SD (mV)	EE (%)
	Code level	mg/mL	Code level	mg/mL	Code level	mg/mL				
Factorial points										
F1	−1	1	−1	1.5	−1	10	165.8 ± 0.4	0.091 ± 0.044	−36.9 ± 0.8	73.80
F2	+1	3	−1	1.5	−1	10	149.7 ± 5.4	0.330 ± 0.001	−47.2 ± 3.3	49.19
F3	−1	1	+1	4.5	−1	10	192.8 ± 0.7	0.109 ± 0.021	−37.1 ± 1.9	78.48
F4	+1	3	+1	4.5	−1	10	249.4 ± 1.1	0.510 ± 0.170	−25.2 ± 9.4	56.91
F5	−1	1	−1	1.5	+1	18	150.1 ± 1.7	0.102 ± 0.043	−34.8 ± 0.5	80.53
F6	+1	3	−1	1.5	+1	18	122.5 ± 5.3	0.194 ± 0.016	−36.6 ± 2.1	53.60
F7	−1	1	+1	4.5	+1	18	183.9 ± 3.8	0.094 ± 0.024	−34.6 ± 0.6	84.43
F8	+1	3	+1	4.5	+1	18	104.6 ± 1.4	0.189 ± 0.042	−29.8 ± 1.4	71.15
Axial points										
F9	1.68	3.68	0	3	0	14	105.2 ± 1.8	0.267 ± 0.057	−37.2 ± 1.4	41.50
F10	−1.68	0.32	0	3	0	14	166.0 ± 3.9	0.101 ± 0.025	−30.9 ± 0.3	90.79
F11	0	2	1.68	5.52	0	14	144.8 ± 3.1	0.281 ± 0.015	−42.1 ± 0.9	47.98
F12	0	2	−1.68	0.48	0	14	110.8 ± 2.6	0.120 ± 0.048	−35.9 ± 0.9	55.60
F13	0	2	0	3	1.68	20.72	115.5 ± 1.2	0.091 ± 0.017	−35.8 ± 0.1	64.57
F14	0	2	0	3	−1.68	7.28	85.5 ± 2.4	0.201 ± 0.039	−34.7 ± 2.8	51.58
Center points										
F15	0	2	0	3	0	14	90.2 ± 1.2	0.078 ± 0.039	−33.6 ± 1.6	56.64
F16	0	2	0	3	0	14	94.4 ± 4.1	0.184 ± 0.123	−34.2 ± 2.6	56.07

**Table 2:** Optimal Formulation DCF NPs.

Factor	Concentration (mg/mL)
[DCF]	1.2
[Lutrol]	3.0
[PLGA]	16.0

**Table 3:** Physicochemical characterization of DCF NPs before and after γ-radiation.

Physicochemical Parameter	Before γ-Radiation	After γ-Radiation
Z_av_ (nm)	149.0 ± 1.4	149.2 ± 0.8
PI	0.060 ± 0.013	0.077 ±0.005
EE (%)	82.4 ± 0.3	80.9 ± 1.8
ZP (mV)	−39.3 ± 1.6	−38.6 ± 2.2

**Table 4:** The half maximal IC_50_ DCF, empty NPs and studied DCF NPs against selected cancer cell lines and non-tumorigenic human breast epithelial cell line (MCF-10A).

Compound	Cell Lines IC_50_ [μg/mL]				
	MV4-11	A-549	MDA-MB-468	MCF-7	MCF-10A
DCF	23.95 ± 5.9	31.02±6.4	33.4 ± 1.8	30.46 ± 7	37.26 ± 1.08
Empty NPs	n.a.	n.a.	n.a.	n.a.	n.a.
DCF NPs	25.74 ± 4.41	69.75 ± 9.4	n.a.	80.6 ± 10	n.a.

Data are presented as mean ± standard deviation (SD) calculated using Prolab-3 system based on Cheburator 0.4 software. n.a.—not active in the range concentration (0.1 μg/mL–100 μg/mL).

## Supplementary Materials

The following supporting information can be downloaded at: https://www.mdpi.com/article/10.3390/pharmaceutics15010102/s1. Figure S1. Stability studies of DCF NPs at 25 °C. (A) Tubiscan backscattering profile, (B) Turbiscan Transmittance profile. Figure S2. Collection of CAM images at 0, 24 h and 48 h after being exposed to NaCL, DCF or DCF NPs.
