# Supplementary material for: Diclofenac Loaded Biodegradable Nanoparticles as Antitumoral and Antiangiogenic Therapy
Source: Pharmaceutics. 2022 Dec 28;15(1):102. doi: 10.3390/pharmaceutics15010102 (PMC9866337; doi:10.3390/pharmaceutics15010102)
Supplement: Supplementary file 1 [file pharmaceutics-15-00102-s001.zip › pharmaceutics-2091242-Supplementary.pdf]

pharmaceutics

MDPI

Supplementary Materials: Diclofenac loaded biodegradable nanoparticles as antitumoral and antiangiogenic therapy

Gerard Esteruelas, Eliana B. Souto, Marta Espina, María Luisa García, Marta Świtalska, Joanna Wietrzyk, Anna Gliszczyńska and Elena Sánchez-López

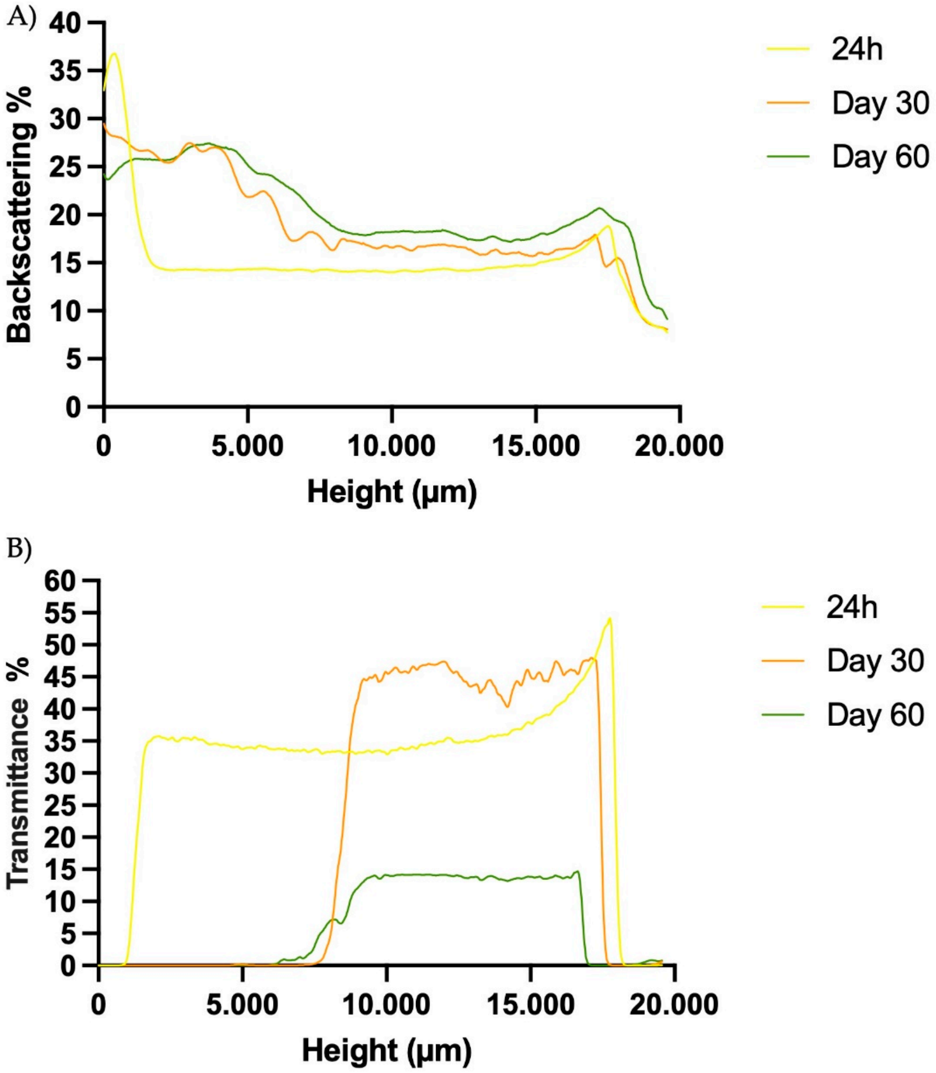

Figure S1. Stability studies of DCF NPs at 25 °C. (A) Tubiscan backscattering profile, (B) Turbiscan Transmittance profile.

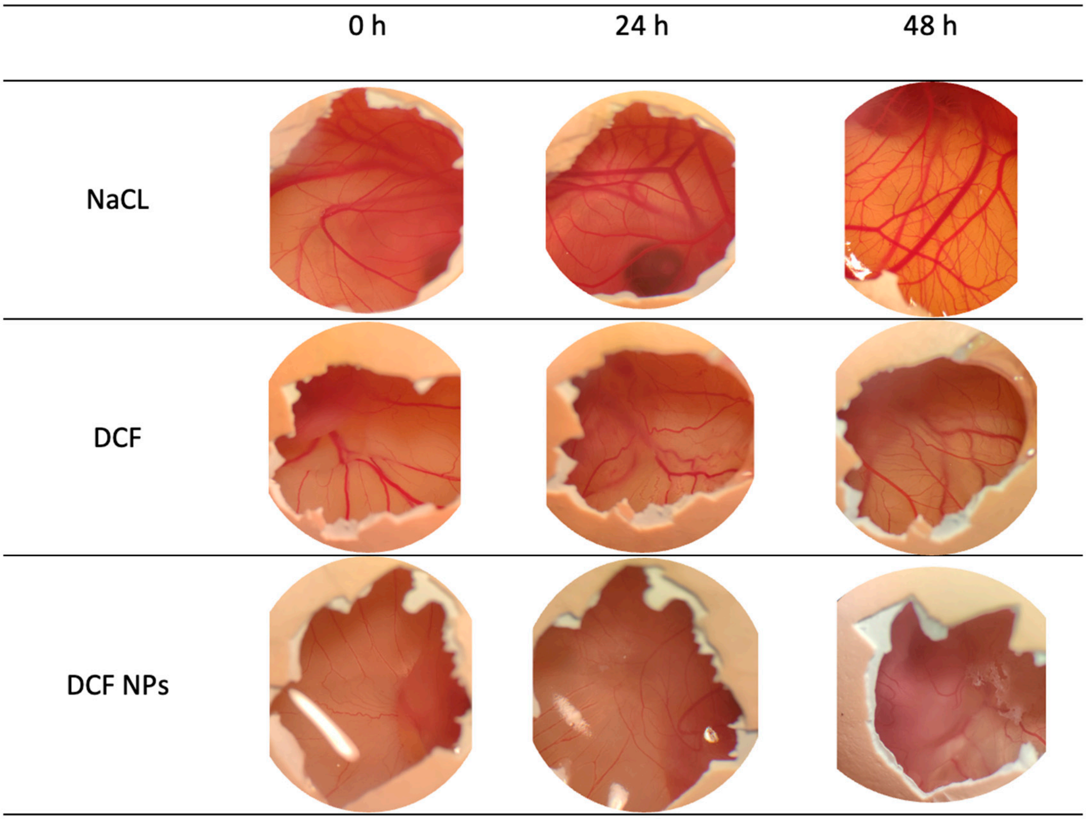

Figure S2. Collection of CAM images at 0, 24 h and 48 h after being exposed to NaCL, DCF or DCF NPs.
